# The effect of guardians' health literacy on the child's spending time at home: A cross-sectional study among Japanese schoolchildren

**DOI:** 10.3934/publichealth.2023005

**Published:** 2023-02-08

**Authors:** Yuki Sato, Reiko Suzuki, Michiko Shigihara, Chieko Suzuki

**Affiliations:** 1 Occupational Stress and Health Management Research Group, National Institute of Occupational Safety and Health, Kanagawa & Tokyo, Japan; 2 Department of Food and Nutrition, Faculty of Human Sciences and Design, Japan Women's University, Tokyo, Japan; 3 Division of Health and Nutrition, Tohoku Seikatsu Bunka University, Sendai, Japan; 4 Department of Nursing, Faculty of Medicine, Saga University, Saga, Japan

**Keywords:** health literacy, guardian, schoolchildren, time spent at home, Japanese

## Abstract

**Background:**

The contents of children's daily activities and the amount of time spent on them has been directly linked to their health and development. Parental health behavior has also been considered a key factor, and the aim of this study was to determine the relationship between parent/guardian health literacy (HL) and their child's time spent at home by behavioral types. The study was conducted in elementary schools in Japan.

**Method:**

The target subjects for this study were elementary schoolchildren (all grades, aged 6 to 12 years) and their parents/guardians, and almost 3000 schoolchildren and their parents/guardians in the Northern and Southern districts in Japan participated. The questionnaire for parents/guardians included amount of time spent per day on the seven major behavioral contents of their child's time at home, on weekdays and weekends, respectively, and a shortened five-item health literacy (HL) scale. Parent/guardian HL results were categorized into two groups (low HL group and high HL group), and we analyzed the association between the HL and child's time spent at home by behavioral contents.

**Results:**

Children in the high HL parent/guardian group spent significantly less time watching TV and playing games than those in the low HL group, both on weekdays and weekends. Time spent playing outside on weekdays and on hobbies on weekdays and weekends was significantly longer for children in the high HL parent/guardian group than in the low HL group. Results of logistic regression analyses adjusted for confounders showed that higher parental/guardian HL reduced children's spending more than 30 minutes watching TV or playing games and increased children's spending more than 30 minutes on outside playing and doing hobbies.

**Conclusions:**

Parental/guardian HL affected the child's time spent at home. The results could suggest that increasing parental/guardian HL has strong potential to improve children's major lifestyle behaviors．

## 1. Introduction

There are still many issues related to children's health, such as lack of time for physical activity, increased time with screens, sedentary time and unbalanced diets [1]–[3]. It has been reported that a healthy lifestyle in childhood is associated with disease risk and mental risk in adulthood [4], and many studies have been conducted on how to promote and acquire healthy lifestyles from childhood [5]. Children's healthy lifestyles and health behaviors have been known to be affected by various factors [6],[7]. Family environment and parents/guardians are influential on children's lifestyles [5],[8], of which parental health literacy is likely to be a strongly relevant key factor [6],[9],[10]. Because children depend on their parents and guardians to prevent and deal with health problems, this suggests that children may be disadvantaged if their parents' and guardians' knowledge and skills, that is, their health literacy, are inadequate [6]. Although there are previous reports on parents'/guardians' health literacy and children's physical activity, nutrient status and screen time, it seems that reports on other patterns of behaviors and the amount of time children spend at home are limited. Therefore, this study examined the association between children's typical lifestyle behaviors, spending time at home and health literacy of their parent/guardian by a cross-sectional study.

## 2. Materials and methods

### 2.1. Study design and subjects

We conducted a cross-sectional questionnaire survey on the lifestyle of schoolchildren and their guardians between November 2015 and March 2016. Nine public elementary schools covering all grades of children (grades 1st to 6th, aged 6–12) in regional central cities in Northern and Southern districts in Japan participated, with a total of 3327 guardians among 4263 enrollees (cooperation rate: 78.0%).

### 2.2. Questionnaires

The question about time spent at home by the child asked for average times (in minutes) per day on a usual day and noted separately weekends and weekdays. The following 7 items were included: watching television, including DVDs and/or another video; playing video games (including handy-type); studying, including homework; reading books (including comic book reading, except for homework); help with family and housework; outside playing; and time doing hobbies.

To estimate health literacy (HL), we used a validated questionnaire with five items, which was short and adapted to the Japanese population [11]. The questions asked about the degree to which a person (i) can gather information about one's own illness and health from various sources, such as newspapers, books, television and the internet; (ii) can pick out the information one needs from lots of information; (iii) can understand the information and communicate it to others; (iv) can judge the credibility of the information; and (v) can decide on plans and actions to improve one's own health based on the information. The structures of these questions are based on communicative HL for the first three questions (items i–iii) and critical HL for the latter two (items iv–v). Each item was rated on a 5-point Likert scale ranging from 1 (“strongly disagree / not at all”) to 5 (“strongly agree”), where high points mean high literacy. The points were summed, and the total scores ranged from a minimum of 5 to a maximum of 25 points.

The questionnaire also contained basic characteristics of the parent/guardian (position from child's side, age, type of employment) and family environment (number of family, number of children, source of household income, feeling of financial leeway). As school policy limited detailed questions such as child's age, school grade, sex/gender and parents'/guardians' personal status, such as marital status, educational background, home economics and other social indicators, alternative minimum questions were adopted in this study.

### 2.3. Statistical analysis

We excluded imperfect responses for child's time spent and items of health literacy, and 3188 individual data were used in analyses (4.2% of the participants excluded). Variables are presented as mean ± standard deviation for continuous variables or prevalence (%) for categorical variables. The total HL score was classified into two categories based on the median or average score. This classification has been adopted in similar previous studies for Japanese people [12]–[14]. In this study, the cut-off score was 18, with less than 18 as the low group (low HL) and 18 or more as the high group (high HL).

We used the chi-square test for comparisons of proportions and Welch's t-test for continuous variables between two groups (low HL, high HL). The distribution of the data showed that a 30-minute interval was appropriate. We also calculated the odds ratio (OR) and the 95% confidence intervals (95% CI) using logistic regression analysis for each category of child time spent in the high HL group at 30 minutes or more with less than 30 minutes as a reference. Adjusting variables included the following: position of parent/guardian from the child's side, age group of parent/guardian, type of employment of parent/guardian, number of family members, number of children, major source of household income, feeling of financial leeway.

All statistical analyses were performed using SPSS version 25 for Windows (IBM Corp., Chicago, IL, USA). The level of statistical significance for each analysis was set at *P* < 0.05.

### 2.4. Ethics

This survey was conducted according to the Ethical Guidelines for Epidemiological Studies established by the Ministry of Health, Labor and Welfare & Ministry of Education, Culture, Sports, Science and Technology in Japan. The Ethics Committee Tohoku University Graduate School of Medicine approved the research protocol (No. 2015–1–810, 2019–1–482). The survey was anonymous, and the submission of the questionnaire was regarded as consent to participate.

## 3. Results

Table 1 shows the background characteristics of parents/guardians and family environments. The overall characteristics of the parents/guardians who participated in this study were the following: Most were mothers (95.2%), and most were in their 30s–40s (47.4%). The most common type of employment was part-time (46.6%), and the next most common was housewife (23.1%).

The most common number of family members was 3–5 (83.1%), with two children (49.9%) being the most common. The main source of household income was a full-time work-based company salary (71.1%), and almost half (49.5%) of the respondents answered that they felt they did not have enough financial leeway. Comparing the characteristics of the HL groups, significant differences were found in the dispersion trends for position from the child's side, number of family members, major source of household income and feeling of financial leeway.

Table 2 shows the results of comparison of child time spent in minutes (min) for the seven behavioral categories by parent/guardian HL group. The child's time spent on watching television was longer in the group with low HL on both weekdays and weekends, with statistically significant differences between the two groups (weekday: low HL group 90.3 min, high HL group 83.2 min, difference 7.07 min, P < 0.001; similarly, weekend: 146.9 min, 136.3 min, difference 10.67 min, P = 0.001). The child's time spent on playing video games was also longer in the group with low HL on both weekdays and weekends, with statistically significant differences between the two groups (weekday: low HL group 34.7 min, high HL group 30.1 min, difference 4.62 min, P < 0.001; similarly, weekend: 70.5 min, 62.0 min, difference 8.51 min, P = 0.001). There were no significant differences between HL groups for time spent studying, reading books or helping with family/housework. The tendency was for time of studying and helping family/housework to be slightly longer in the higher HL group and time of reading books to be slightly longer in the lower HL group. The child's time spent on playing outside was greater in the high HL group both on weekdays and on weekends. A statistically significant difference was shown only on weekdays (low HL group 28.8 min, high HL group 32.3 min, difference 3.50 min, P < 0.001). The child's time spent on doing hobbies was greater in the high HL group on both weekdays and weekends, with statistically significant difference (weekday: low HL group 8.4 min, high HL group 10.0 min group, difference 1.64 min, P < 0.038; similarly, weekend: 17.8 min, 21.6 min, difference 3.74 min, P = 0.014).

**Table 1.** Demographic characteristics among parents/guardians according to HL groups.

		Total (n = 3188)	Parent/guardian HL score		P*
			Low group (n = 1689)	High group (n = 1499)	
**Position from child's side**				
	Mother	95.2	94.6	95.8	0.035
	Father	3.5	3.8	3.2	
	Others	0.8	1.2	0.4	
**Age group, years old**				
	<30	2.4	2.1	2.7	0.351
	30–39	47.4	47.0	47.8	
	40–49	47.2	47.7	46.7	
	>49	2.5	2.8	2.2	
**Type of employment**				
	Full time job	22.0	23.1	22.5	0.114
	Part time job	46.6	43.4	45.1	
	Self-employed	4.0	4.9	4.4	
	Housewife	23.1	24.5	23.8	
	On leave of absence/parental leave	0.9	1.4	1.2	
	Seeking employment	0.4	0.4	0.4	
	Others	2.4	1.3	1.9	
**Number of family members**				
	<3 persons	2.4	2.8	1.8	0.003
	3–5 persons	83.1	81.1	85.5	
	>5 persons	14.1	15.7	12.1	
	Unknown	0.5	0.4	0.6	
**Number of children**				
	1 child	15.0	14.8	15.3	0.896
	2 children	49.9	49.8	50.0	
	>2 children	35.1	35.4	34.8	
**Major source of household income**				
	Self-employed (including agriculture, forestry, and fisheries)	7.6	7.7	7.4	0.045
	Company employee, full-time salary	71.1	72.2	70.0	
	Company employee, part-time salary	12.6	11.1	14.2	
	Public servant salary	5.1	4.9	5.3	
	Others	0.4	0.5	0.3	
**Feeling of financial leeway**				
	Enough	26.6	22.9	30.9	<0.001
	Not enough	49.5	51.5	47.4	
	Neither	22.8	24.3	21.1	

*Note: *Comparisons of variance between groups of HL; “Unknown” responses were removed from the table; total % is less than 100 in some places.

**Table 2.** Contents and minutes of child's time spent at home compared by HL groups.

			Parent/guardian HL score		Difference*	*P* ^#^
			Low group	High group		
**Contents of daily spending time**		
	**Watching television**	
		Weekday	90.3±57.4	83.2±54.3	7.07	<0.001
		Weekend	146.9±94.5	136.3±90.2	10.67	0.001
	**Playing video games**	
		Weekday	34.7±39.6	30.1±34.4	4.62	<0.001
		Weekend	70.5±72.1	62.0±68.0	8.51	0.001
	**Studying**	
		Weekday	49.2±25.7	49.3±25.4	−0.11	0.902
		Weekend	42.1±39.0	43.3±35.5	−1.22	0.353
	**Reading books**	
		Weekday	18.2±22.6	17.6±19.4	0.64	0.389
		Weekend	26.9±32.9	26.1±30.8	0.83	0.463
	**Helping with family/housework**	
		Weekday	10.5±12.6	11.0±12.2	−0.47	0.280
		Weekend	15.6±20.8	15.8±17.6	−0.19	0.781
	**Playing outside**	
		Weekday	28.8±35.5	32.3±36.6	−3.50	0.006
		Weekend	73.4±85.9	78.0±89.7	−4.65	0.136
	**Doing hobbies**	
		Weekday	8.4±22.6	10.0±22.0	−1.64	0.038
		Weekend	17.8±40.7	21.6±44.7	−3.74	0.014

*Note: Values in table: mean ± standard deviation, values in minutes; *Difference of the mean of the low-scoring group minus the mean of the high-scoring group; ^#^Welch t-test.

**Table 3.** Results of odds ratios of time spent by children with high HL group parents/guardians.

			No. in HL group		Crude OR (95%CI)	*P*	Adjusted OR (95%CI)	*P*
			Low	High				
**Watching television**				
	Weekday	<30 min	87	107	1.00 (ref.)		1.00 (ref.)	
		≥30 min	1602	1392	0.71 (0.53–0.95)	0.020	0.71 (0.53–0.95)	0.022
	Weekend	<30 min	104	108	1.00 (ref.)		1.00 (ref.)	
		≥30 min	1585	1391	0.85 (0.64–1.12)	0.237	0.86 (0.65–1.14)	0.304
**Playing video games**		
	Weekday	<30 min	729	701	1.00 (ref.)		1.00 (ref.)	
		≥30 min	960	798	0.86 (0.75–0.99)	0.041	0.86 (0.75–0.99)	0.038
	Weekend	<30 min	413	440	1.00 (ref.)		1.00 (ref.)	
		≥30 min	1276	1059	0.78 (0.67–0.91)	0.002	0.78 (0.66–0.91)	0.002
**Studying**					
	Weekday	<30 min	165	133	1.00 (ref.)		1.00 (ref.)	
		≥30 min	1520	1362	1.11 (0.87–1.41)	0.387	1.11 (0.87–1.41)	0.396
	Weekend	<30 min	474	378	1.00 (ref.)		1.00 (ref.)	
		≥30 min	1214	1121	1.16 (0.99–1.36)	0.068	1.16 (0.99–1.36)	0.064
**Reading books**					
	Weekday	<30 min	1111	988	1.00 (ref.)		1.00 (ref.)	
		≥30 min	576	511	1.00 (0.86–1.16)	0.974	0.99 (0.86–1.15)	0.904
	Weekend	<30 min	903	779	1.00 (ref.)		1.00 (ref.)	
		≥30 min	786	720	1.06 (0.92–1.22)	0.399	1.05 (0.91–1.21)	0.482
**Helping with family/housework**		
	Weekday	<30 min	1465	1296	1.00 (ref.)		1.00 (ref.)	
		≥30 min	223	203	1.03 (0.84–1.26)	0.784	1.05 (0.86–1.29)	0.632
	Weekend	<30 min	1280	1124	1.00 (ref.)		1.00 (ref.)	
		≥30 min	409	375	1.04 (0.89–1.23)	0.600	1.06 (0.90–1.25)	0.486
**Playing outside**		
	Weekday	<30 min	864	683	1.00 (ref.)		1.00 (ref.)	
		≥30 min	825	816	1.25 (1.09–1.44)	0.002	1.24 (1.08–1.43)	0.003
	Weekend	<30 min	524	425	1.00 (ref.)		1.00 (ref.)	
		≥30 min	1165	1074	1.14 (0.98–1.32)	0.100	1.14 (0.97–1.33)	0.105
**Doing hobbies**		
	Weekday	<30 min	1445	1208	1.00 (ref.)		1.00 (ref.)	
		≥30 min	244	291	1.43 (1.18–1.72)	<0.001	1.39 (1.15–1.68)	0.001
	Weekend	<30 min	1270	1049	1.00 (ref.)		1.00 (ref.)	
		≥30 min	419	450	1.30 (1.11–1.52)	0.001	1.27(1.09–1.49)	0.003

*Note: Abbreviations in the table: OR = odds ratio, CI = confidence interval, ref. = reference; Adjustment variables as follows: position of parent/guardian from child's side, age group of parent/guardian, type of employment of parent/guardian, number of family members, number of children, major source of household income, feeling of financial leeway.

Table 3 shows the odds ratios for child time spent for more than 30 minutes compared to less than 30 minutes in the group with high parental/guardian HL. The ORs of spending more than 30 minutes for watching television and video game playing were lower in the higher HL group. The adjusted ORs were 0.71 (95% CI = 0.53–0.95, P = 0.002) for weekday television watching, 0.86 (95% CI = 0.75–0.99, P = 0.038) for weekday video game playing and 0.78 (95% CI = 0.66–0.91, P = 0.002) for weekend video game playing. The ORs were close to 1.00 for study time, reading time and helping with family/housework either on weekdays or weekends. The ORs of spending more than 30 minutes playing outside and doing hobbies were higher in the higher HL group. The adjusted ORs were 1.24 (95% CI = 1.08–1.43, P = 0.003) for weekday playing outside, 1.39 (95% CI = 1.15–1.68, P = 0.001) for weekday hobby time and 1.27 (95% CI = 1.09–1.49, P = 0.003) for weekend hobby time. In a detailed analysis, significant differences in weekday television watching, weekday playing video games, weekend playing video games and weekday outside playing were even more noticeable for the more than 120 minutes time (not shown in the result table). The adjusted ORs were 0.65 (95% CI = 0.46–0.92, P = 0.041) for weekday television watching, 0.64 (95% CI = 0.47–0.88, P = 0.006) for weekday playing video games, 0.67 (95% CI = 0.55–0.82, P < 0.001) for weekend playing video games and 1.46 (95% CI = 1.05–2.04, P = 0.026) for weekday outside playing.

## 4. Discussion

We examined the association between parental/guardian health literacy and child's time spent at home, based on data of approximately 3000 Japanese schoolchildren. Overall, parental/guardian HL was strongly associated with the following four behavioral categories of time spent by children: watching television, playing video games, playing outside and doing hobbies. Our results for TV time and game time are consistent with previous studies, and regarding outdoor play as a physical activity, the direction of the results of the present study agrees with other previous studies [1],[3],[6]–[8].

The largest difference in terms of number of hours was found for weekend watching television, which was approximately 10.6 minutes/day longer in the low parent/guardian HL group. The second largest time difference was found for weekend time playing video games, which was about 8.5 minutes/day longer in the low parent/guardian HL group. Children in the high parent/guardian HL group spent approximately 4.6 minutes more time playing outside on weekends and 3.7 minutes more time doing hobbies on weekends than children in the low parent/guardian HL group. The differences were measured in minutes and appeared small; however, this difference would be larger with long-term cumulation. Elementary schools in Japan have roughly 200 school days in a year. It is estimated that a difference of 7 min/day in children's weekday TV watching amounts to a difference of 1400 min/year, that is, about 23 hours.

The results of the logistic analysis indicated that higher parental/guardian HL may reduce the probability of children spending more than 30 minutes watching TV on weekdays and playing games on weekdays and weekends. Higher parent/guardian HL indicated that the probability of children spending more than 30 minutes outside on weekdays and on weekday and weekend hobbies increased. Sub-analyses also suggested that higher parental/guardian HL, less likely for children to spend more than 2 hours watching TV on weekdays and playing games on weekdays and weekends while more likely for children to spend more than 2 hours playing outside. These results suggest that if parents/guardians have a high level of HL, it might be possible to reduce the amount of time children spend watching TV and playing games to less than 30 minutes or less than 120 minutes and to increase the amount of time they spend playing outside and enjoying their hobbies to more than 30 minutes or more than 120 minutes.

In recent years, reports have also accumulated on children's screen time, sedentary time and their health disadvantages [3],[5],[15],[16]. The TV watching and video gaming time collected in this study could be substituted as total screen time or sitting time, although use of computers, mobile phones and smartphones was not included. Hence, screen time and sitting time were not estimated in this study. Our study also has some limitations. First, the data on children's time spent at home was based on questionnaire responses by parents/guardians and not actual measurement data, so some response errors were unavoidable. There may also be differences in content-specific time by child age and by sex. Our present study has not fully examined the variables. The information such as the child's sex/gender, year of age and class could not be added to the questionnaire due to concerns about the possibility of identifying the child. In particular, the time spent playing outside may differ by gender and age group. This remains an issue for future surveys. It has been also reported that parenting style could be significantly related to children's daily life and health behaviors [17]–[20]. The present study may not have adequately adjusted for parental parenting attitudes. It may also be insufficient for SES indicators, but the impact of SES as a predictor of health literacy appears to be limited [21].

The present study focused on the relationship between parental/guardian HL and seven behavioral categories of child's time spent at home. There are few reports investigating the time spent by Japanese schoolchildren and the parents' HL, so the results of this study are very meaningful. Many factors, including the parental/guardian HL focused on in this study, have direct and indirect or combinatory influences on children's health behaviors and health outcomes. Further findings are expected from comprehensive and continuing epidemiological study.

## 5. Conclusions

Our study conducted in Japanese schoolchildren showed that parental/guardian HL was associated with some contents of child's time spent at home. High parental/guardian HL was negatively associated with children's time spent watching TV and playing games, while times spent playing outside and doing hobbies were positively associated.
